# Global research status and trends of enteric glia: a bibliometric analysis

**DOI:** 10.3389/fphar.2024.1403767

**Published:** 2024-05-24

**Authors:** Huai-Yu Li, Wei-Xin Yan, Jia Li, Jing Ye, Zhi-Guo Wu, Zheng-Kun Hou, Bin Chen

**Affiliations:** ^1^ Department of Gastroenterology, The First Affiliated Hospital of Guangzhou University of Chinese Medicine, Guangzhou, China; ^2^ Guangdong Clinical Research Academy of Chinese Medicine, Guangzhou, China; ^3^ The First Clinical College, Guangzhou University of Chinese Medicine, Guangzhou, China; ^4^ School of Clinical Medicine, Jiangxi University of Chinese Medicine, Nanchang, China; ^5^ Clinical Medical College of Acupuncture, Moxibustion and Rehabilitation, Guangzhou University of Chinese Medicine, Guangzhou, China

**Keywords:** enteric glial cells, enteric nervous system, bibliometric analysis, purinergic signaling, inflammation, intestinal motility, gut microbiota

## Abstract

**Background:**

Enteric glia are essential components of the enteric nervous system. Previously believed to have a passive structural function, mounting evidence now suggests that these cells are indispensable for maintaining gastrointestinal homeostasis and exert pivotal influences on both wellbeing and pathological conditions. This study aimed to investigate the global status, research hotspots, and future directions of enteric glia.

**Methods:**

The literature on enteric glia research was acquired from the Web of Science Core Collection. VOSviewer software (v1.6.19) was employed to visually represent co-operation networks among countries, institutions, and authors. The co-occurrence analysis of keywords and co-citation analysis of references were conducted using CiteSpace (v6.1.R6). Simultaneously, cluster analysis and burst detection of keywords and references were performed.

**Results:**

A total of 514 publications from 36 countries were reviewed. The United States was identified as the most influential country. The top-ranked institutions were University of Nantes and Michigan State University. Michel Neunlist was the most cited author. “Purinergic signaling” was the largest co-cited reference cluster, while “enteric glial cells (EGCs)” was the cluster with the highest number of co-occurring keywords. As the keyword with the highest burst strength, Crohns disease was a hot topic in the early research on enteric glia. The burst detection of keywords revealed that inflammation, intestinal motility, and gut microbiota may be the research frontiers.

**Conclusion:**

This study provides a comprehensive bibliometric analysis of enteric glia research. EGCs have emerged as a crucial link between neurons and immune cells, attracting significant research attention in neurogastroenterology. Their fundamental and translational studies on inflammation, intestinal motility, and gut microbiota may promote the treatment of some gastrointestinal and parenteral disorders.

## 1 Introduction

The primary regulation of gastrointestinal function is controlled by the enteric nervous system (ENS), which consists of a complex neuronal and glial network, mainly involving the myenteric plexus located in the muscular layer and the submucosal plexus below the mucosal layer (Sharkey and Mawe, 2023). The importance of enteric neurons in intestinal function has been extensively studied (Rhee et al., 2009), yet our current understanding falls short of fully elucidating the control mechanism of intestinal reflex. Recently, there has been a growing focus on the involvement of enteric glia in intestinal homeostasis, specifically regarding their relationship with the cell bodies and processes of enteric neurons.

The glia in the ENS were first described by Dogiel in the late 19th century (Dogiel, 1899) and were defined as Schwann cells based on their anatomical characteristics. It was not until the 1970s that Gabella discovered that glial cells found in the gastrointestinal tract have small central perikarya and stellate processes that were phenotypically similar to astrocytes and named them “enteric glial cells (EGCs)” for the first time in 1981 (Gabella, 1972; Gabella, 1981). The downstream effects of chronically activated EGCs are generally beneficial. Historically, EGCs were considered peripheral glia that contribute to the structural and nutritional protection of enteric neurons. In recent studies, EGCs have been confirmed to be an essential node in intestinal tissue circuitry, regulating gastrointestinal motility, mucosal barrier integrity, immune response, neurogenesis, and neuroprotection through dynamic interactions with intestinal epithelial cells, immune cells, and intestinal neurons (Gulbransen and Sharkey, 2012). The dense structure of EGCs is not limited to the ganglion range in the ENS, and they are widely distributed throughout the intestinal wall. Based on their morphological and anatomical location within the intestinal wall, EGCs exhibit local heterogeneity in specific gastrointestinal regions, with their phenotype determined by the unique microenvironment of the gastrointestinal tract (Coelho-Aguiar Jde et al., 2015). EGCs located in the mucosa directly below epithelial cells influence epithelial and intestinal barrier function, while EGCs in the submucosal or intermuscular plexus embed neurons and regulate neurotransmission. Once intestinal homeostasis fails, reactive EGCs respond to any pathophysiological disturbances by changing their molecular composition, structure, and/or function, depending on the severity and type of injury, and in severe cases, trigger reactive gliosis. These findings challenge the conventional notion that EGCs merely serve as passive support cells and revolutionize our understanding of the pathogenesis of dysfunction in common intestinal diseases such as inflammatory bowel disease (IBD) (Ochoa-Cortes et al., 2016), irritable bowel syndrome (IBS) (Aziz et al., 2021), and colorectal cancer (Garcia et al., 2014). In addition, reactive EGCs are also regarded as exacerbating factors for certain neurological disorders involving the gastrointestinal tract (Seguella et al., 2021; Claudino Dos Santos et al., 2023). Comprehending the field of enteric glia may provide potential research avenues for the development of neurogastroenterology and new therapeutic strategies for gastrointestinal and parenteral diseases.

Bibliometrics, initially introduced by Pritchard in 1969, involves the objective analysis of the structure, quantity, and patterns of change within scientific literature information (Pritchard, 1969). The scientific knowledge map functions as a visual representation of scientific knowledge, facilitating the examination of research trends and hotspots within a specific field across temporal and spatial dimensions (Shiffrin and Börner, 2004). Although much progress has been achieved in glial biology, this field remains relatively nascent as an emerging discipline. Over the past decade, growing interest in the hypothesis that enteric glia are actively involved in intestinal function has led to a rapid increase in the literature describing the properties of these cells, yet no bibliometric analysis related to this field has been published. Therefore, we adopted bibliometric methods and scientific knowledge maps to visually analyze articles collected in the Web of Science Core Collection (WoSCC) from 2003 to 2022, aiming to provide comprehensive data and references on the global research status, emerging areas of interest, and future trends of enteric glia.

## 2 Materials and methods

### 2.1 Search strategy

**FIGURE 1:**
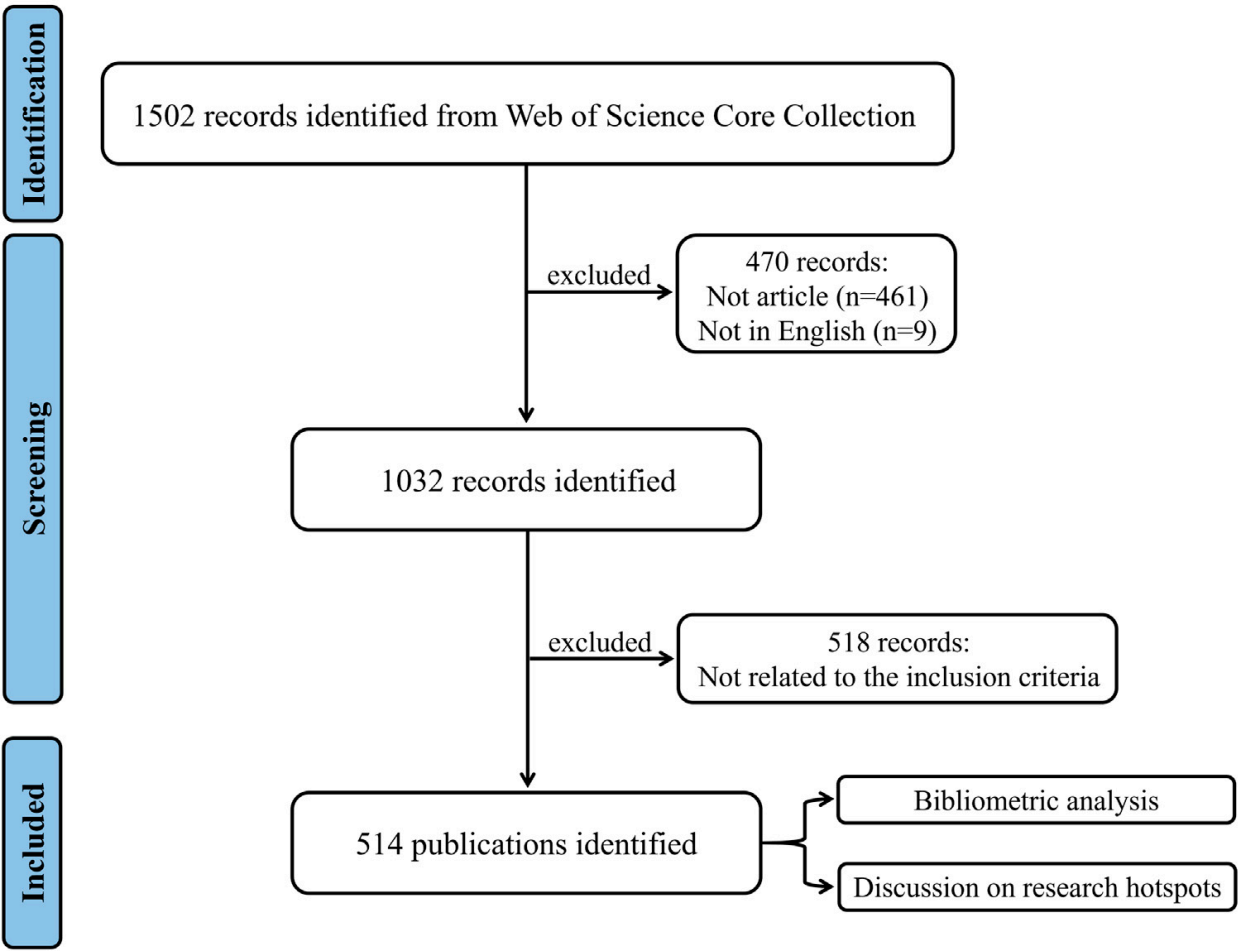
Flowchart of the literature screening process.

The data were obtained from the Science Citation Index-Expanded (SCI-Expanded) of WoSCC, covering published literature from 1 January 2003, to 31 December 2022. The search strategy was set as follows: TS = ((enteric glia) OR (enteric glial cell$) OR (enteric glia cell$)). The literature type was set to Article, and the language was confined to English. The full records and cited references of all documents were exported in plain text format. After removing duplicates, the title, abstract, and full text of each paper were systematically reviewed to obtain original articles related to enteric glia. The literature search and evaluation were independently conducted by two researchers, with any disagreement resolved through discussion with a third party. The process of bibliographic retrieval and collection is illustrated in Figure 1.

### 2.2 Data processing

In this study, Origin (version 2021), R (version 4.1.2), VOSviewer (version 1.6.19) (van Eck and Waltman, 2010), and CiteSpace (version 6.1.R6) (Chen, 2006) were used for data analysis and bibliometric visualization. Origin was used to perform statistical analysis on the annual publication and citation counts of the selected valid literature. The co-operation analysis of countries/institutions/authors was conducted using VOSviewer, while the national geographical distribution map of enteric glia studies was displayed using R. We merged keywords with the same meaning based on our relevant expertise. The co-occurrence analysis of keywords and the co-citation analysis of references were conducted using CiteSpace. Additionally, cluster analysis and burst detection were performed for both keywords and references. The log-likelihood rate method was used as clustering algorithm, and the clusters generated in CiteSpace were tagged with keywords. Cluster analysis aims to reveal key directions in development trends, and burst detection enables the identification of abrupt surges in research interest within a specific field over a particular timeline.

## 3 Results

### 3.1 The trends in global publications outputs

**FIGURE 2:**
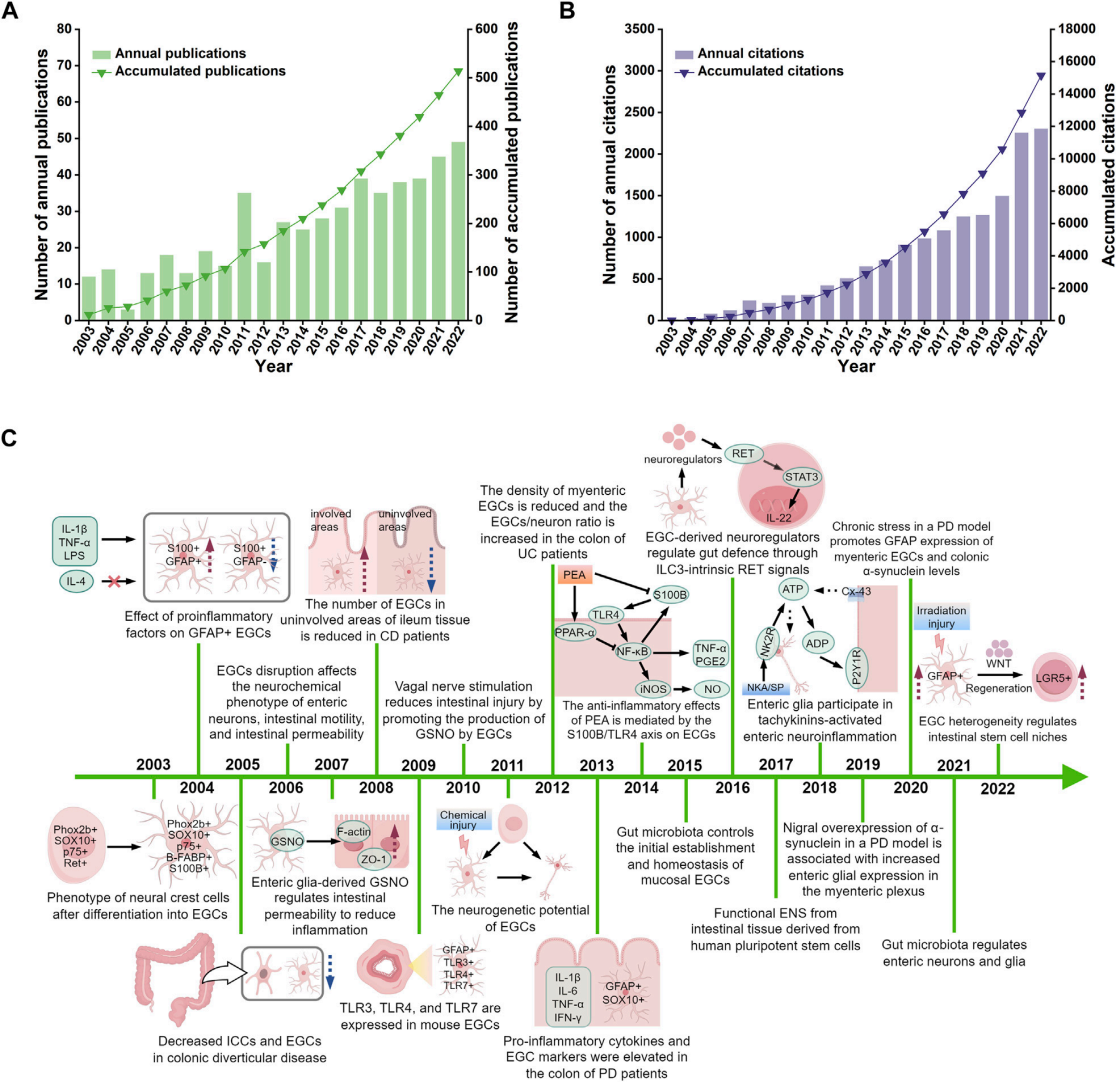
The distribution of publications **(A)**, their citations **(B)**, and highly cited studies **(C)** from 2003 to 2022. IL-1β, interleukin-1beta; TNF-α, tumour necrosis factor-alpha; LPS, lipopolysaccharide; IL-4, interleukin-4; GFAP, glial fibrillary acidic protein; EGCs, enteric glial cells; ICCs, interstitial cells of cajal; GSNO, s-nitrosoglutathione; ZO-1, zonula occludens-1; TLR-3, toll-like receptor-3; TLR-4, toll-like receptor-4; TLR-7, toll-like receptor-7; IL-6, interleukin-6; IFN-γ, interferon-gamma; PD, Parkinson’s disease; CD, Crohns disease; UC, ulcerative colitis; PEA, palmitoylethanolammide; PPAR-α, peroxisome proliferator-activated receptor-alpha; NF-κB, nuclear factor kappa-B; iNOS, inducible nitric oxide synthase; NO, nitric oxide; PGE2, prostaglandin E2; IL-22, interleukin-22; NKA, neurokinin A; SP, substance P; NK2R, neurokinin-2 receptor; ATP, adenosine triphosphate; ADP, adenosine diphosphate; Cx43, connexin-43; LGR5, leucine-rich repeat-containing G-protein coupled receptor 5. Figure 2C was drawn by Figdraw.

The 514 relevant original articles were selected from 1,502 literature records collected by WoSCC. The number of annual publications in the field of enteric glia increased steadily from 2003 to 2010, representing its early stage. Between 2011 and 2022, there was a significant rise in the number of annual publications, with the cumulative number of publications in 2022 being 4.8 times that of 2010 (Figure 2A). Compared to the period before 2019, the annual number of citations between 2020 and 2022 showed a qualitative leap (Figure 2B). These data indicate that enteric glia had emerged as a prominent and extensively researched topic globally during this period. Based on the number of citations, Supplementary Table S1 shows the top 10 highly-cited papers in the field of enteric glia. The paper titled “*Colonic inflammation in Parkinson’s disease*” (Devos et al., 2013) published in 2013 received the most citations, and the paper titled “*Engineered human pluripotent-stem-cell-derived intestinal tissues with a functional enteric nervous system*” (Workman et al., 2017) published in 2017 had the highest annual average number of citations. The subjects addressed in the annually most cited publications are shown in Figure 2C. These studies redefine the fundamental role and molecular mechanisms of enteric glia in the ENS (Young et al., 2003; Laranjeira et al., 2011; Workman et al., 2017; Baghdadi et al., 2022) and the microbiota-gut-brain (MGB) axis (Costantini et al., 2010; Kabouridis et al., 2015; Vicentini et al., 2021), emphasizing their therapeutic potential in several gastrointestinal motility disorders (Bassotti et al., 2005; Aubé et al., 2006), neuroinflammatory conditions (von Boyen et al., 2004; Savidge et al., 2007; Villanacci et al., 2008; Barajon et al., 2009; Bernardini et al., 2012; McClain et al., 2014; Brown et al., 2016; Delvalle et al., 2018a), and neurodegenerative diseases (Devos et al., 2013; Dodiya et al., 2020; O'Donovan et al., 2020).

### 3.2 Distribution of countries/regions, institutions and authors

**FIGURE 3:**
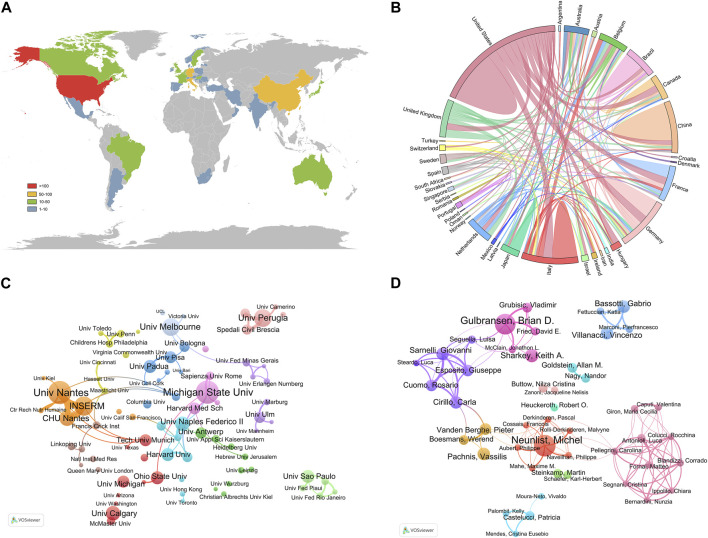
Countries/regions, institutions, and authors contributing to the research of enteric glia from 2003 to 2022. **(A)** The distribution of publications of different countries/regions. **(B)** The cooperation relationship between countries/regions. **(C)** The network visualization map of productive institutions. **(D)** The network visualization map of productive authors.

Relevant studies on enteric glia involved a total of 36 countries/regions, mainly distributed in North America, Asia, and Europe (Figure 3A). Among them, the United States was the most prolific country, with a total of 164 papers published, followed by Italy (78) and China (76) (Table 1). The H-index is a hybrid quantitative indicator of academic achievement (Hirsch, 2005). As depicted in Table 1, the United States (44) had the highest H-index, signifying its predominant influence on enteric glia research, followed by Germany (29) and Italy (27). In addition, the United States exhibited the highest level of international collaboration, primarily with Italy, Germany, France, and Canada, highlighting its bridging role in facilitating breakthrough achievements (Figure 3B).

**TABLE 1:** Top 10 prolific countries/regions of enteric glia from 2003 to 2022.

Rank	Country	Counts	Percentage	Total citations	H-index	TLS
1	United States	164	31.91	6325	44	117
2	Italy	78	15.18	2846	27	48
3	China	76	14.79	1288	22	20
4	Germany	62	12.06	2613	29	54
5	Brazil	48	9.34	750	17	18
6	France	47	9.14	2944	26	36
7	United Kingdom	36	7.00	2372	25	46
8	Canada	33	6.42	1511	21	26
9	Belgium	29	5.64	1587	20	42
10	Australia	24	4.67	1091	16	33

TLS, total link strength.

The institutions with the highest number of publications were University of Nantes (25) and Michigan State University (25), followed by Institut National de la Sante et de la Recherche Medicale (INSERM, 20), of which INSERM was engaged in this field earlier (Table 2). The most significant institutional collaboration network exists between the University of Nantes, INSERM, and CHU de Nantes from France (Figure 3C). Referring to the highly cited articles in collaboration between these three institutions, we discovered that their research mainly focused on the intrinsic association between EGCs and dysfunction in Parkinson’s disease (PD) (Devos et al., 2013; Clairembault et al., 2014) as well as Crohns disease (CD) (Le Loupp et al., 2015; Pochard et al., 2016).

**TABLE 2:** Top 13 productive institutions of enteric glia from 2003 to 2022.

Rank	Institution	Country	Counts	Percentage	Total citations	H-index	TLS
1	University of Nantes	France	25	4.86	1896	20	73
2	Michigan State University	United States	25	4.86	626	14	35
3	INSERM	France	20	3.89	1895	25	72
4	University of Melbourne	Australia	18	3.50	924	15	38
5	University of Perugia	Italy	18	3.50	699	12	40
6	CHU de Nantes	France	15	2.92	1323	19	51
7	State University of Maringa	Brazil	15	2.92	249	10	23
8	University of Naples Federico II	Italy	14	2.72	859	14	24
9	University of Calgary	Canada	14	2.72	800	12	15
10	Harvard University	United States	12	2.33	949	16	32
11	University of Padua	Italy	12	2.33	523	11	31
12	Ohio State University	United States	12	2.33	444	10	17
13	University of Sao Paulo	Brazil	12	2.33	185	8	13

TLS, total link strength.

As shown in Table 3, the authors with the most publications were Michel Neunlist from INSERM (23) and Brian D. Gulbransen from Michigan State University (23). Michel Neunlist had the highest H-index (19), followed by Brian D. Gulbransen (14) and Carla Cirillo (13). Among the author co-operation network, there existed the closest collaboration between Brian D. Gulbransen and Keith A. Sharkey, as well as Vincenzo Villanacci and Gabrio Bassotti (Figure 3D). These authors have made significant contributions to the understanding of the specific role played by enteric glia.

**TABLE 3:** Top 10 productive authors of enteric glia from 2003 to 2022.

Rank	Author	Institution	Counts	Citation	H-index	TLS
1	Michel Neunlist	INSERM	23	2199	19	195
2	Brian D. Gulbransen	Michigan State University	23	766	14	97
3	Keith A. Sharkey	University of Calgary	14	800	12	71
4	Gabrio Bassotti	University of Perugia	14	436	11	105
5	Vassilis Pachnis	Francis Crick Institute	13	1263	12	91
6	Carla Cirillo	Katholieke Universiteit Leuven	13	824	13	97
7	Giovanni Sarnelli	University of Naples Federico II	13	797	12	120
8	Vincenzo Villanacci	Spedali Civili di Brescia	13	642	11	107
9	Pieter Vanden Berghe	Katholieke Universiteit Leuven	12	927	12	68
10	Rosario Cuomo	University of Naples Federico II	12	818	12	111

TLS, total link strength.

### 3.3 Journals analysis

Publications related to enteric glia were published in 216 academic journals. A total of 150 articles were published in the top 10 active journals, representing 29.18% of total publications (Supplementary Table S2). According to Bradford’s Law (Brookes, 1969), the top 10 journals with the most published publications are considered core journals in this field. They can serve as the preferred choice for relevant researchers to submit and access articles related to enteric glia. *Neurogastroenterology and Motility* (42) had the highest number of publications, followed by *American Journal of Physiology-Gastrointestinal and Liver Physiology* (19) and *Gastroenterology* (19). The journal with the highest H-index was *Neurogastroenterology and Motility* (22), followed by *Gastroenterology* (18) and *American Journal of Physiology-Gastrointestinal and Liver Physiology* (16).

### 3.4 Analysis of co-cited references

**FIGURE 4:**
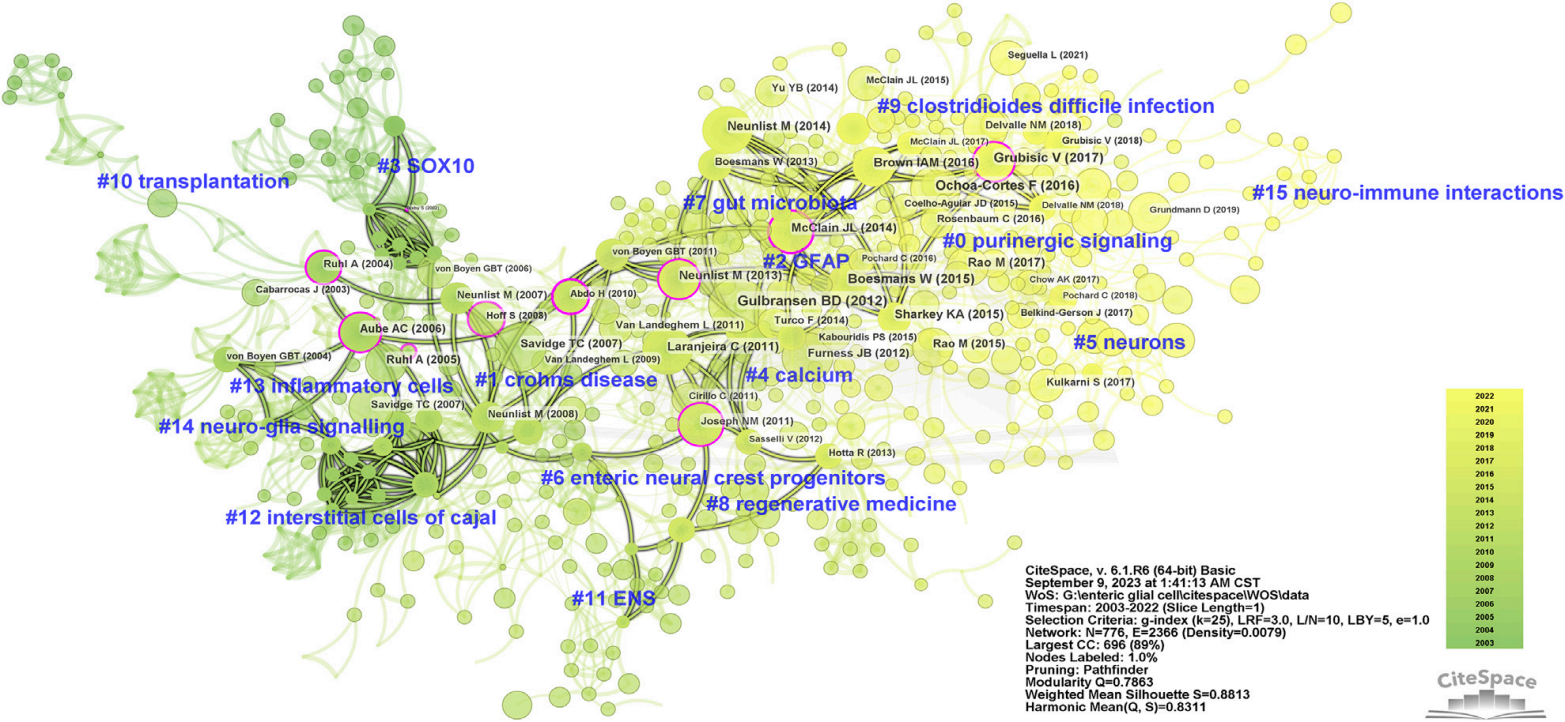
The knowledge map of co-cited references related to enteric glia from 2003 to 2022. GFAP, glial fibrillary acidic protein; ENS, enteric nervous system.

There is a co-citation relationship between different references cited by the same publication, and the frequency of co-citations reflects its value as a theoretical basis in a certain field (Small, 1973). We summarized the top 10 co-cited references of enteric glia (Table 4). Among them, the paper “*Novel functional roles for enteric glia in the gastrointestinal tract*” (Gulbransen and Sharkey, 2012) published by Brian D. Gulbransen in 2012 had the highest number of co-citations. All co-cited references were constructed into a knowledge map containing 16 clusters with diverse labels, which intuitively revealed the evolution of the knowledge base of enteric glia research over time (Figure 4). According to the results of cluster analysis, “purinergic signaling” (cluster #0) was the largest cluster in this field. The bidirectional communication of purinergic signals between enteric glia and neurons offers a novel avenue for investigating the regulation of gastrointestinal reflexes and neuroinflammatory processes. “SOX10” (cluster #3) and “transplantation” (cluster #10) were the early knowledge base of enteric glia research, while “regenerative medicine” (cluster #8), “gut microbiota” (cluster #7), “purinergic signaling” (cluster #0), “clostridioides difficile infection” (cluster #9), “neurons” (cluster #5), and “neuro-immune interactions” (cluster #15) reflected the research direction of the current stage.

**TABLE 4:** Top 10 co-cited references of enteric glia from 2003 to 2022.

Rank	Title	First author	Journal	Co-citation	Year
1	*Novel functional roles for enteric glia in the gastrointestinal tract* (Gulbransen and Sharkey, 2012)	Brian D. Gulbransen	Nat Rev Gastroenterol Hepatol	47	2012
2	*Enteric glia: the most alimentary of all glia* (Grubišić and Gulbransen, 2017a)	Vladimir Grubisic	J Physiol-London	40	2017
3	*Heterogeneity and phenotypic plasticity of glial cells in the mammalian enteric nervous system* (Boesmans et al., 2015)	Werend Boesmans	Glia	37	2015
4	*Enteric Glial Cells: A New Frontier in Neurogastroenterology and Clinical Target for Inflammatory Bowel Diseases* (Ochoa-Cortes et al., 2016)	Fernando Ochoa-Cortes	Inflamm Bowel Dis	35	2016
5	*Enteric glial cells: recent developments and future directions* (Neunlist et al., 2014)	Michel Neunlist	Gastroenterology	31	2014
6	*Ca2+ responses in enteric glia are mediated by connexin-43 hemichannels and modulate colonic transit in mice* (McClain et al., 2014)	Jonathon L. McClain	Gastroenterology	31	2014
7	*Enteric glia mediate neuron death in colitis through purinergic pathways that require connexin-43 and nitric oxide* (Brown et al., 2016)	Isola A M Brown	Cell Mol Gastroenterol Hepatol	31	2016
8	*Enteric Glia Regulate Gastrointestinal Motility but Are Not Required for Maintenance of the Epithelium in Mice* (Rao et al., 2017)	Meenakshi Rao	Gastroenterology	30	2017
9	*Emerging roles for enteric glia in gastrointestinal disorders* (Sharkey, 2015)	Keith A. Sharkey	J Clin Invest	29	2015
10	*Glial cells in the mouse enteric nervous system can undergo neurogenesis in response to injury* (Laranjeira et al., 2011)	Catia Laranjeira	J Clin Invest	27	2011

In addition, we identified the top 15 references based on the strength of citation burst (Kleinberg, 2003) (Supplementary Figure S1). The first citation burst involved “*Enteric glia*” (Rühl et al., 2004) by Anne Ruhl in 2004. The reference with the strongest burst was “*Novel functional roles for enteric glia in the gastrointestinal tract*” (Gulbransen and Sharkey, 2012), followed by “Tor C. Savidge, 2007” (Savidge et al., 2007) and “Anne Ruhl, 2005” (Rühl, 2005). Until 2022, “Fernando Ochoa-Cortes, 2016” (Ochoa-Cortes et al., 2016), “Isola A. M. Brown, 2016” (Brown et al., 2016), “Vladimir Grubisic, 2017” (Grubišić and Gulbransen, 2017a), and “Meenakshi Rao, 2017” (Rao et al., 2017) still showed strong burst strength, indicating that these studies are hot events in the knowledge base and worthy of further exploration.

### 3.5 Keywords analysis

**FIGURE 5:**
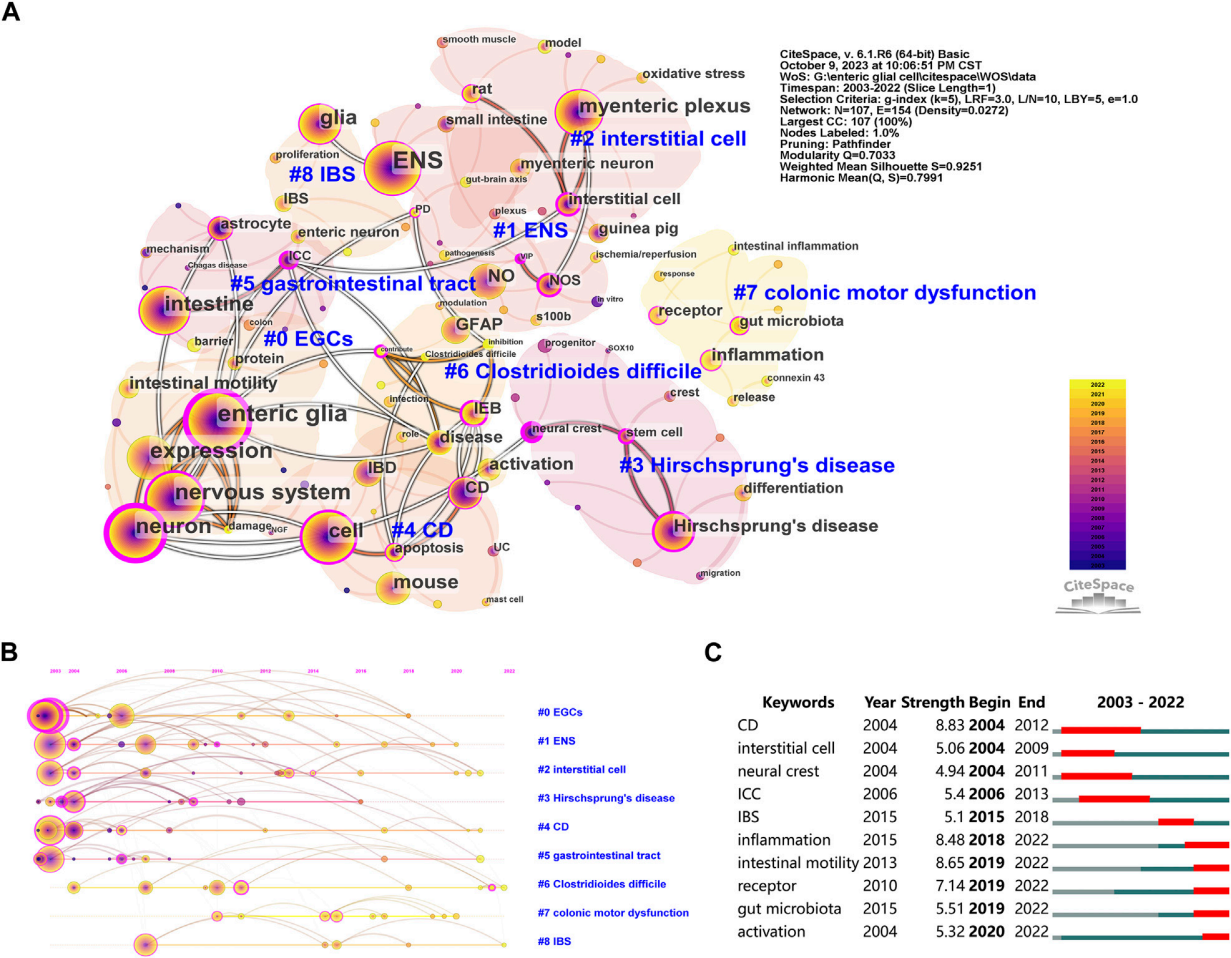
The co-occurrence map **(A)**, timeline view **(B)**, and burst detection **(C)** of keywords related to enteric glia from 2003 to 2022. EGCs, enteric glial cells; ENS, enteric nervous system; NO, nitric oxide; GFAP, glial fibrillary acidic protein; IEB, intestinal epithelial barrier; IBD, inflammatory bowel disease; CD, Crohns disease; IBS, irritable bowel syndrome; NOS, nitric oxide synthase; ICC, interstitial cell of cajal; UC, ulcerative colitis; PD, Parkinson’s disease; VIP, vasoactive intestinal peptide; NGF, nerve growth factor.

The keyword co-occurrence analysis helps identify research hotspots in enteric glia and predict future research directions. We merged similar keywords in 514 documents and generated a keyword co-occurrence map of 107 nodes and 9 clusters (Figure 5A). Table 5 lists the disease keywords by co-occurrence frequency, among which the top three diseases identified were Hirschsprung’s disease, CD, and IBS. Sorted by the number of keywords, the largest cluster was “EGCs” (cluster #0), including keywords such as “enteric glia,” “nervous system,” “neuropathy,” “intestinal motility,” and “NF-κb”. Followed by “ENS” (cluster #1), which included keywords such as “myenteric neuron,” “EGF,” “ischemia/reperfusion,” “S100B,” “NOS,” and “colitis”. The keyword co-occurrence map reveals the presence of multiple overlapping clusters, suggesting a strong interrelation among these topics. According to the range of years, the clusters of enteric glia were divided into two stages: keywords in “EGCs” (cluster #0), “gastrointestinal tract” (cluster #5), “Hirschsprung’s disease” (cluster #3), “CD” (cluster #4), “ENS” (cluster #1), and “interstitial cell” (cluster #2) were more common in the early stages of the field, while keywords in “Clostridioides difficile” (cluster #6), “IBS” (cluster #8), and “colonic motor dysfunction” (cluster #7) may be the focus of recent research (Figure 5B).

**TABLE 5:** Top 8 disease keywords of enteric glia from 2003 to 2022.

Rank	Disease keyword	Frequency
1	Hirschsprung’s disease	40
2	Crohns disease	31
3	Irritable bowel syndrome	23
4	Ulcerative colitis	9
5	Parkinson’s disease	8
6	Intestinal ischemia/reperfusion	7
7	Clostridioides difficile	5
8	Chagas disease	4

Furthermore, Figure 5C lists the top 10 keywords with the strongest burst. The first keywords with burst strength were “CD”, “interstitial cell” and “neural crest”. The top three keywords with the strongest burst were “CD” (8.83), “intestinal motility” (8.65), and “inflammation” (8.48). It is worth noting that the keywords “inflammation,” “intestinal motility,” “receptor,” “gut microbiota,” and “activation” were in burstiness from 2019 to the present, indicating their potential as emerging frontiers in enteric glia studies.

## 4 Discussion

### 4.1 General trends

To better understand the current status of enteric glia research, this study examined 514 relevant original articles in the WoSCC database over the past 20 years (2003–2022) based on bibliometrics. The rapid growth in publications and citations indicate that enteric glia as an emerging focus is receiving sustained attention from scholars around the world. We analyzed the most influential countries, institutions, and authors in the field of enteric glia. The total number of publications from the United States, Italy, and China accounted for more than half of the total, among which the United States, as the country with the highest number of publications, influence, and level of cooperation in this field, had cooperative relations with 21 countries/regions, and the closest cooperation with Italy. Although China ranked third in the world in terms of the total number of publications, its level of international cooperation was relatively inadequate. In the institutional collaboration network, University of Nantes and INSERM from France and Michigan State University from the United States were the top 3 institutions with the number of publications. Notably, most collaborations of the publications were from domestic organizations, with a noticeable absence of institutional collaboration across different countries. Therefore, strengthening exchanges and cooperation between countries and institutions is particularly important to promote further development. Michel Neunlist from INSERM and Brian D. Gulbransen from Michigan State University were the most active authors in this field. As the most influential author, Michel Neunlist was mainly engaged in the intrinsic connection between EGCs and the pathogenesis of PD (Devos et al., 2013; Clairembault et al., 2014) and CD (Le Loupp et al., 2015; Pochard et al., 2016), while Brian D. Gulbransen focused on exploring how EGCs participate in neuronal regulation of intestinal motility (Gulbransen and Sharkey, 2009; McClain et al., 2014; Delvalle et al., 2018b) and how they regulate immune cells to affect intestinal inflammation (Grubišić et al., 2020; Chow et al., 2021).

In addition, we identified the top 10 articles of enteric glia, most of which focus on the mechanism of EGCs in neuro-immune interactions or inflammation. The most cited paper was published in *Neurobiology of Disease* by David Devos in 2013, which innovatively found increased expression levels of enteric glial markers glial fibrillary acidic protein (GFAP) and SOX10 in the colon of PD patients and proposed a new theory that EGCs may be involved in the occurrence and development of PD and related intestinal dysfunction (Devos et al., 2013). The paper with the highest average annual citations was published in Nature Medicine in 2017 Workman et al. (2017). The study was the first to link human pluripotent stem cell-derived neural crest cells with functional ENS to human intestinal organoids and successfully differentiate into new neurons and glial cells in the intestinal mesenchyme (Workman et al., 2017). It may provide a new treatment model for diseases with enteric glia as their pathologic basis.

### 4.2 Knowledge base

The analysis of co-cited references was used to investigate the research foundation in the field of enteric glia. Based on the number of co-citations, we identified high-impact references in enteric glia research, which represent the main focus of researchers in the knowledge base. The top 10 most-cited references included 5 reviews and 5 original articles. These 5 reviews summarized and supplemented the new findings of enteric glia in intestinal motility, neuron-glia communication and intestinal epithelial barrier (IEB) in different years (2012–2017), and emphasized that its functional performance in enteric neuro-immune interactions and some enteral and parenteral diseases. Laranjeira et al. (2011) found that enteric glia and neurons in the mouse intestine were derived from neural crest precursor cells expressing the transcription factor Sox10. The neurogenic potential of these cells was highest in the second trimester and gradually disappeared between 1 and 3 months after birth. Interestingly, they also found that most mature EGCs continued to maintain Sox10 expression in adult mice and acquired the ability to generate functional enteric neurons *in vitro* culture and *in vivo* after enteric ganglion injury (Laranjeira et al., 2011). These findings provided a novel theoretical framework for the treatment of diseases related to intestinal neuron degeneration (such as IBD) and diseases related to intestinal neuron loss (such as Hirschsprung’s disease). McClain et al. (2014) were the first to demonstrate that connexin-43 hemichannels mediated purinergic signaling-induced Ca^2+^ responses in mouse colon EGCs, connexin-43 ablation resulted in Ca^2+^ wave propagation disorder in the glial network, consequently leading to delayed colonic transit and reduced isometric force generation in the intestine. This study offered significant molecular insights for targeting enteric glia to regulate gastrointestinal motility. By using Mosaic Analysis with Double Markers, fate mapping, lineage tracing, and Ca^2+^ imaging, Boesmans et al. (2015) showed that EGCs in mammals exhibited different molecular and functional characteristics based on differences in morphology and location and had a certain degree of phenotypic plasticity, which suggests that the unique microenvironment of the intestinal wall may be a determinant of the phenotype of EGCs and its typical biomarker expression. The data of Brown et al. (2016) confirmed that enteric glia promoted neuroinflammation and subsequent neuronal death. They found that intestinal neuroinflammation activated neuronal P2X7 receptors and led to the release of neuronal adenosine triphosphate (ATP) in pannexin 1 channels, which was then rapidly hydrolyzed to adenosine diphosphate (ADP) by eNTPDase2 to activate glial P2Y1 receptors and glial cell activity in the gut. They also found that glial P2Y1 receptors enhanced connexin-43 hemichannel-dependent ATP release by driving inducible nitric oxide synthase (iNOS) to produce nitric oxide (NO), thereby promoting neuronal death and neuroinflammatory processes (Brown et al., 2016). Rao et al. (2017) used the proteolipid protein 1 promoter to deplete mouse enteric glia. They found that, in addition to increasing the frequency of colonic migrating motor complexes (CMMCs) in female mice, it did not lead to changes in intestinal epithelial proliferation, intestinal barrier, and intestinal neuron survival. These results argued against the necessity of EGCs to maintain IEB integrity and survival of enteric neurons under homeostatic conditions and were consistent with Grubišić and Gulbransen (2017b).

In order to explore the hot papers in the knowledge base of enteric glia, we also screened out the top 15 references with the strongest citation bursts, including the top 10 references with the most citations. “Isola A. M. Brown (2016)” (Brown et al., 2016), “Fernando Ochoa-Cortes (2016)” (Ochoa-Cortes et al., 2016), “Vladimir Grubisic (2017)” (Grubišić and Gulbransen, 2017a), and “Meenakshi Rao (2017)” (Rao et al., 2017), as high-impact references, remained in citation bursts until 2022, suggesting that clinical and basic research workers in enteric glia need to pay more attention to the research directions involved in these articles. In summary, the above references provide essential theoretical support for the current paradigm of understanding the role of enteric glia in digestive physiology and pathophysiology.

### 4.3 Research hotspots and frontiers

We further analyzed the research hotspots and frontiers in enteric glia through keyword co-occurrence analysis. The keyword co-occurrence map suggests that the understanding of enteric glia function in the past 20 years was mainly focused on neuron-glia communication, intestinal motility, inflammation, and IEB, which were associated with intestinal diseases (Hirschsprung’s disease, IBD, IBS), infectious diseases (Clostridioides difficile infection, Chagas disease) and parenteral diseases. It is worth pointing out that CD in IBD, as the keyword with the strongest burst, was one of the hot topics in early enteric glia research. Based on tissue biopsy, researchers found that the gliosis in the inflammatory area of intestinal mucosa in patients with CD was far less evident than that in patients with UC, and the EGC density in uninvolved intestinal mucosal areas was lower than that in the control group (Cornet et al., 2001; von Boyen et al., 2011). The above evidence suggests that CD may be a form of enteric gliopathy associated with disruption of the glial cell network. The studies describing the involvement of EGCs in the pathogenesis of CD highlight their cellular properties in the IEB, glia-immune interactions, and neuroplasticity. These processes are expected to drive the development of further therapeutic strategies for CD. In addition, the results of the burst detection of keywords revealed that “inflammation,” “intestinal motility,” “receptor,” “gut microbiota,” and “activation” are among the prominent areas in enteric glia research, reflecting recent advancements in this field. Here, discussion is focused on the potential of enteric glia in inflammation, intestinal motility, and gut microbiota.

#### 4.3.1 Inflammation

The resolution of inflammation is necessary for the maintenance of mucosal integrity. Based on glial ablation animal models, earlier studies evaluating the functional significance of the glial network in intestinal homeostasis found a functional link between enteric glia and inflammation. For example, the depletion of enteric glia in transgenic mice expressing thymidine kinase gene of the herpes simplex virus (HSV-Tk) from the mouse GFAP promoter (Bush et al., 1998) and in autoimmune mice targeting CD8^+^ T cells (Cornet et al., 2001) led to the rapid development of fulminant jejuno-ileitis and even death. Although the absence of glia in both models is associated with severe colonic inflammation, unknown methodological effects on intestinal epithelium, neurons, and immune cells diminish the importance of the data obtained from these glial ablation animal models. Ablation of enteric glia also obscures a clear understanding of the cellular properties of specific subtypes, and more selective methods are needed to elucidate the role of glial signaling in the ENS homeostasis.

What is clear is that EGCs are actively involved in intestinal inflammation in a variety of diseases, such as IBD, postoperative ileus (POI), and bacterial and viral infections, from which they derive a “reactive EGC phenotype”. Depending on the severity and type of inflammation, its location in the intestine, and the glial signals transmitted, the context-dependent and disease-specific enteric glial network responds to pathological perturbations by changing its molecular expression, structure, and/or function, and in severe cases reactive enteric gliosis occurs. The current assessment of reactive EGCs relies on the expression of biomarkers such as GFAP and S100β, as well as morphological changes. Notably, nuclear factor kappa-B (NF-κB) signaling associated with increased S100β, as the primary pathway of pro-inflammatory stimuli in EGCs, has been proven to be associated with dextran sodium sulphate induced colitis (Esposito et al., 2014), enteroinvasive *Escherichia coli* infection (Turco et al., 2014), UC(Cirillo et al., 2009; Esposito et al., 2014), human immunodeficiency virus-1-induced diarrhea (Sarnelli et al., 2018), and colon carcinoma (Seguella et al., 2019; Seguella et al., 2020). Additionally, the potential of enteric glia S100β in promoting NF-κB transcription and sequestrating pro-apoptotic wild-type p53 is considered an ideal bridge between mucosal inflammation and colorectal cancer, necessitating further investigation (Seguella et al., 2019; Seguella et al., 2020).

By releasing damaging and/or protective factors, reactive EGCs interact with the nervous and immune systems to exert a dual modulatory action in intestinal inflammation (Ochoa-Cortes et al., 2016). For example, EGCs-derived NO is a key mediator of neurodegeneration (Brown et al., 2016) and epithelial barrier dysfunction (MacEachern et al., 2015) during intestinal inflammation, with its production being tightly regulated by toll-like receptor (TLR) (Turco et al., 2014) and S100β/receptor for advanced glycation end products (RAGE) signaling pathways (Cirillo et al., 2009). Specifically, the overproduced S100β in reactive EGCs enters the cell through binding with RAGE on the surface of EGCs. Subsequently, it interacts with MyD88 downstream of TLR, leading to NF-κB activation and translocation into the nucleus. This process drives the upregulation of iNOS activity and triggers downstream release of NO. Furthermore, conclusive data confirm that the improvement of peroxisome proliferator-activated receptor-alpha (PPAR-α) agonist palmitoylethanolamide in UC and colitis mouse models is attributed to its inhibitory effect on NO production in EGCs. This finding suggests that targeting NO production in the enteric glia may be a potential new therapeutic strategy for colitis (Esposito et al., 2014). In addition, extracellular ATP, functioning as a purinergic component actively involved in neurogenic inflammation, serves as a bidirectional messenger facilitating communication between intestinal neurons and EGCs. Through pannexin 1 channels, neurons release high concentrations of the neurotransmitter ATP to drive neurogenic inflammation and stimulate glial cell activity encoded by intracellular Ca^2+^ responses. In turn, the Ca^2+^ response of enteric glia triggers the release of the gliotransmitter ATP through NO-controlled connexin-43 hemichannels, thereby inducing the continuation and amplification of neurogenic inflammation (Gulbransen et al., 2012). Some evidence suggests that P2X7 receptor (P2X7R) in the ENS are involved in the pathophysiology of intestinal inflammation. However, the location of P2X7R is still controversial. Earlier studies assumed that P2X7R in enteric neurons is involved in neuronal cell death and colitis-related motor dysfunction (Gulbransen et al., 2012; Brown et al., 2016). In contrast, a recent study pointed out that P2X7R is mainly expressed in macrophages and S100β-positive EGCs and denied the idea that P2X7R exists in enteric neurons (Jooss et al., 2023). In a short phase IIa clinical study (Eser et al., 2015), the oral P2X7R antagonist AZD9056 reduced abdominal pain in patients with moderate to severe CD, indicating that the blocking of P2X7R contributed to the reduction of visceral pain. However, P2X7R blocking resulted in an increased incidence of colitis-related cancers in mice (Hofman et al., 2015). Therefore, the efficacy and adverse effects of long-term use of P2X7R antagonists need to be further evaluated in subsequent studies.

Interestingly, despite exhibiting enhanced pro-inflammatory function during intestinal inflammation, reactive EGCs release factors such as glial-derived neurotrophic factor (GDNF)(Zhang et al., 2010), glial-derived s-nitrosoglutathione (GSNO) (Savidge et al., 2007), 15-hydroxyeicosatetraenoic acid (Pochard et al., 2016), and 11β-prostaglandinF2α (Coquenlorge et al., 2016) that contribute to the preservation of intestinal barrier integrity. This suggests that reactive EGCs may represent an endeavor to preserve intestinal homeostasis and could be associated with the functional status of specific subtypes of EGC. Additionally, the activation of adenosine A_2B_ receptors on EGCs contributes to the release of GDNF, substance P, and IL-1β, thereby reducing intestinal inflammation associated with obesity induced by high fat diet (Antonioli et al., 2020; D'Antongiovanni et al., 2020). The specific mechanism of enteric glial-immune interactions in intestinal inflammation remains poorly understood. Some data show that reactive EGCs actively participate in intestinal immune response by expressing immune response-related genes and secreting inflammatory mediators. They also play a regulatory role in both innate and adaptive immune cells, including macrophages (Grubišić et al., 2020), type 3 innate lymphoid cells (ILC3) (Ibiza et al., 2016), NK cells (Progatzky et al., 2021), and T cells (Cornet et al., 2001; Kermarrec et al., 2016). Therefore, restoring normal EGC function may be an effective strategy to suppress inflammation and may be a clinical target for intestinal inflammatory diseases such as IBS.

#### 4.3.2 Intestinal motility

Abnormalities in intestinal motility are broadly defined as failure to maintain local homeostasis in the ENS, including EGCs. A study from 1985 initially proposed that EGCs are involved in the regulation of intestinal motility based on the phenomenon of diarrhea caused by the degeneration of EGCs in suckling mice (Aikawa and Suzuki, 1985). The pivotal role of neurons in the ENS has long been established, while the beneficial impact of EGCs on intestinal motility has only recently started to be unraveled. With the visualization of Ca^2+^ imaging (Gulbransen and Sharkey, 2009; Boesmans et al., 2019), researchers have discovered that neuronal excitation during intestinal movement facilitates the diffusion of EGCs activity. In contrast to neurons that generate action potentials, the excitability of EGCs responsible for sensing neuronal activity is determined by intracellular Ca^2+^ responses and transmitted between cells through Ca^2+^ signals, Ca^2+^ oscillations or Ca^2+^ waves in enteric neural circuits. In GFAPhM3Dq transgenic “DREADD” (a designer receptor activated only by the designer drug) mice, selective activation of the Gq-coupled receptors dependent Ca^2+^ response in GFAP-positive EGCs induced neurogenic smooth muscle contractions, CMMCs, and colonic motility, suggesting that Ca^2+^ signaling to enteric glia enhances motor neurocircuits within the gastrointestinal tract (McClain et al., 2015). The neural regulation of intestinal motility relies on the coordination of neuron-to-glia communication, in which EGCs activity encoded by the intracellular Ca^2+^ response is stimulated by the release of neurotransmitters involved in fast excitatory neurotransmission, such as ATP (McClain et al., 2014), acetylcholine (Delvalle et al., 2018b), and serotonin (Okamoto et al., 2014). The Ca^2+^ response of EGCs subsequently triggers the release of gliotransmitters, including ATP (Brown et al., 2016) and GABA (Fried et al., 2017), which modulate intestinal nerve reflexes. Furthermore, the involvement of EGCs in intestinal motility, particularly their interactions with non-neuronal cell types such as interstitial cells of Cajal within the intestinal wall, poses a significant question for future research regarding its impact on intestinal reflexes.

The reduction of EGCs not only contributes to the impairment of neuron-glia communication, but also compromises their ability to support neuronal survival and promote neurogenesis, all of which are crucial factors in the development of intestinal dysmotility. Current evidence suggests that a decrease in EGCs is associated with reduced intestinal motility (Aubé et al., 2006; Nasser et al., 2006; Rao et al., 2017) and linked to disturbances in movement patterns observed in individuals with slow-transit constipation, diverticular disease, Chagas disease, and idiopathic megacolon (Bassotti et al., 2005; Bassotti et al., 2006; Iantorno et al., 2007). However, the factors that exacerbate EGCs loss are unclear and appear to be influenced by dietary factors (Baudry et al., 2012) and the process of aging (McClain et al., 2014). The comprehension of the mechanisms by which EGCs facilitate neuronal survival and neurogenesis while maintaining their differentiation status holds significant implications for advancing the treatment of diseases. Additionally, reactive gliosis occurs in constipation associated with PD (Clairembault et al., 2015), POI(Stoffels et al., 2014), and intestinal dysmotility in inflammatory conditions (Ochoa-Cortes et al., 2016), in which manipulation of specific glial signaling mechanisms has a significant effect on intestinal motility. For example, the selective ablation of connexin-43 restricts purinergic signaling in the glial network and weakens colonic transit under normal intestinal reflexes (Brown et al., 2016). The antagonism of interleukin-1 (IL-1) receptor reduces the expression of interleukin-6 (IL-6) and monocyte chemotactic protein 1 in EGCs, thereby preventing local immune cell infiltration and postoperative intestinal obstruction (Stoffels et al., 2014). These EGCs signaling molecules represent potential new therapeutic targets for gastrointestinal motility disorders.

#### 4.3.3 Gut microbiota

Under physiological conditions, the commensal microbiota residing in the intestine assists the absorption of nutrients and prevents the damage of IEB by pathogenic bacteria through competitive inhibition [74]. As an important component of intestinal homeostasis, gut microbiota also contributes to the maintenance of the ENS development and maturation (Kabouridis and Pachnis, 2015; Kayama et al., 2020). Recent data support for the role of gut microbiota in promoting migration of EGCs from the nerve plexus within the intestinal wall to the intestinal mucosa (Kabouridis et al., 2015). Furthermore, it has been observed that depletion of gut microbiota due to antibiotic exposure results in a non-selective loss of intestinal neurons and region-specific reduction in EGCs(Vicentini et al., 2021), thereby raising additional concerns regarding the intricate relationship between gut microbiota and EGCs. The immune tolerance induced by regulatory T cells and anti-inflammatory cytokines enables the commensal microbiota in the gut to evade immune activation, while being closely monitored by host immunity (Kayama et al., 2020). The increased intestinal permeability and barrier dysfunction associated with microbial dysbiosis result in a diminished host tolerance to gut microbiota, in which EGCs are thought to be related to the intestinal immune response against invasive microorganisms. Studies in humans have reported the abnormal colonization of adherent-invasive *E. coli* (AIEC) in individuals with CD (Palmela et al., 2018). Furthermore, the activation of AIEC in EGCs induces TLR/S100B-RAGE dependent NO signaling, which may be an important pathway for CD infection (Turco et al., 2014). Interestingly, EGCs also exert a protective effect in the invasion of intestinal pathogens. For example, data obtained from human colonic explants and intestinal epithelial cells indicate that GSNO reduces damage to the intestinal epithelial barrier and inflammatory response caused by *Shigella* flexneri invasion (Flamant et al., 2011). GDNF also controls neurotrophic factors and innate IL-22 by activating RET in ILC3, thereby regulating mucosal homeostasis and anti-microbial defense of the intestine (Artis and Spits, 2015; Ibiza et al., 2016).

The rapid development of microbiome technology has revolutionized the traditional understanding of the gut-brain (GB) axis into a systematic biological perspective of MGB axis interactions (Mayer et al., 2022). Through the bidirectional action of the GB axis, gut microbiota disorder repeatedly impacts psychological states via central nervous system (CNS) feedback, while emotional stress in turn affects intestinal motility, permeability, and visceral sensitivity (Mayer et al., 2022). The interaction of EGCs with gut microbiota in neuroinflammation and psychiatric disorders has led to a reevaluation of the pathophysiology of several diseases, with a focus on PD (Claudino Dos Santos et al., 2023) and IBS(Jeffery et al., 2012; Meira de-Faria et al., 2021; Han et al., 2022). For example, gastrointestinal manifestations associated with gut microbiota precede motor symptoms and disease diagnosis in PD, accompanied by an upregulation in GFAP expression and phosphorylation levels associated with EGCs (Clairembault et al., 2014). According to the Braak hypothesis, reactive gliosis driven by α-synuclein or other neurotropic agents may contribute to the progression of the progression of PD to the CNS by exacerbating local inflammatory responses and synaptic dysfunction in the gut (Claudino Dos Santos et al., 2023). The prevalence of mood disorders in IBS patients and their general neglect significantly contribute to the poor actual benefit of clinical treatments (Staudacher et al., 2021). Altered gut microbiota may be both a product of IBS pathology and a potential factor in the onset and progression of the disease. microbe-associated molecular patterns of gut microbiota, such as lipopolysaccharides, lipoproteins and/or flagellin, initiate a sustained inflammatory state in the ENS and CNS by contacting pattern recognition receptors in intestinal immune cells or EGCs to trigger the release of local cytokines, ultimately leading to increased visceral pain perception and sensitivity (Aguilera-Lizarraga et al., 2022). Future targets may involve the effects of gut microbiota on specific EGCs, which may be involved in the pathogenesis of GB-related diseases. In conclusion, exploring the role of EGCs interactions with gut microbiota in gut physiology and pathophysiology may unveil novel and intriguing avenues for research.

## 5 Strengths and limitations

In order to visually and objectively present the global status, current trends and future directions of enteric glia research, we conducted a multi-dimensional analysis of relevant papers in WoSCC. Although this is the first bibliometric analysis and literature visualization in this field, our study inevitably has some inherent limitations. First, our study may not have captured all relevant glial research publications, as not all papers were indexed in the WoSCC. As a result, we may have missed some publications due to database bias. Secondly, there is no uniform standard for the parameter settings of CiteSpace and VOSviewer, which may cause deviation in the output results of cluster analysis under different Settings. In addition, constant updating of WoSCC results in slight differences between the final results and the current results, which may exclude some recently published high-quality studies. Nevertheless, with reference to previous similar studies, we must acknowledge these limitations, which do not detract from the importance or contribution of the publications in this study to the field. The findings of our study provide reference values for newcomers to glia research and present more crucial leads and concepts for subsequent investigations.

## 6 Conclusion

This study showed the global status and trends of enteric glia research based on bibliometrics. Although the diversity of enteric glia in intestinal function is only beginning to be revealed, global trends in publications and citations suggest that they have become an active subject of research in neurogastroenterology. The United States dominated the network of cooperation as the most influential country in this field. The analysis of co-cited references and keywords revealed that the research hotspots of enteric glia from 2003 to 2022 mainly focused on neuron-to-glia communication, intestinal motility, inflammation and IEB. The future research directions in this field primarily revolve around three key aspects: 1) understanding the specific mechanisms of enteric glial-immune interactions in intestinal inflammation; 2) identifying the key factors in neuron-glia communication that trigger intestinal dysmotility; 3) to explore the role of enteric glia in the MGB axis and its association with some parenteral diseases. Fundamental and translational investigations in these areas provides new avenues for comprehending gastrointestinal pathophysiology and may complement innovative clinical perspectives towards therapeutic strategies for gastrointestinal and parenteral disorders.

## Data Availability

The datasets presented in this study can be found in online repositories. The names of the repository/repositories and accession number(s) can be found in the article/Supplementary Material.
