# Supplementary material for: Global research status and trends of enteric glia: a bibliometric analysis
Source: Front Pharmacol. 2024 May 24;15:1403767. doi: 10.3389/fphar.2024.1403767 (PMC11157232; doi:10.3389/fphar.2024.1403767)
Supplement: Supplementary file 1 [file Table1.DOCX]

Supplementary Table S1 Top 10 highly-cited papers of enteric glia from 2003 to 2022.

| Rank | Title | First author | Journal | Citation | Average citation per year | Year |
| --- | --- | --- | --- | --- | --- | --- |
| 1 | *Colonic inflammation in Parkinson's disease* | David Devos | Neurobiol Dis | 379 | 37.9 | 2013 |
| 2 | *Engineered human pluripotent-stem-cell-derived intestinal tissues with a functional enteric nervous system* | Michael J Workman | Nat Med | 360 | 60 | 2017 |
| 3 | *Enteric glia regulate intestinal barrier function and inflammation via release of S-nitrosoglutathione* | Tor C. Savidge | Gastroenterology | 305 | 19.06 | 2007 |
| 4 | *Glial cells in the mouse enteric nervous system can undergo neurogenesis in response to injury* | Catia Laranjeira | J Clin Invest | 269 | 22.42 | 2011 |
| 5 | *Glial-cell-derived neuroregulators control type 3 innate lymphoid cells and gut defence* | Sales Ibiza | Nature | 239 | 34.14 | 2016 |
| 6 | *Microbiota Controls the Homeostasis of Glial Cells in the Gut Lamina Propria* | Panagiotis S. Kabouridis | Neuron | 219 | 27.38 | 2015 |
| 7 | *Toll-Like Receptor 2 Regulates Intestinal Inflammation by Controlling Integrity of the Enteric Nervous System* | Paola Brun | Gastroenterology | 200 | 20 | 2013 |
| 8 | *Toll-like Receptors 3, 4, and 7 Are Expressed in the Enteric Nervous System and Dorsal Root Ganglia* | Isabella Barajon | J Histochem Cytochem | 192 | 13.71 | 2009 |
| 9 | *Palmitoylethanolamide improves colon inflammation through an enteric glia/toll like receptor 4-dependent PPAR-alpha activation* | Giuseppe Esposito | Gut | 187 | 20.78 | 2014 |
| 10 | *Acquisition of neuronal and glial markers by neural crest-derived cells in the mouse intestine* | Heather M. Young | J Comp Neurol | 180 | 9 | 2003 |
