# Supplementary material for: Global research status and trends of enteric glia: a bibliometric analysis
Source: Front Pharmacol. 2024 May 24;15:1403767. doi: 10.3389/fphar.2024.1403767 (PMC11157232; doi:10.3389/fphar.2024.1403767)
Supplement: Supplementary file 2 [file Table2.DOCX]

Supplementary Table S2 Top 10 active journals of enteric glia from 2003 to 2022.

| Rank | Journal | Counts | Citation | H-index | IF（2023） |
| --- | --- | --- | --- | --- | --- |
| 1 | *Neurogastroenterology and Motility* | 42 | 1239 | 22 | 3.5 |
| 2 | *American Journal of Physiology-Gastrointestinal and Liver Physiology* | 19 | 918 | 16 | 4.5 |
| 3 | *Gastroenterology* | 19 | 1725 | 18 | 29.4 |
| 4 | *Plos One* | 14 | 583 | 12 | 3.7 |
| 5 | *Scientific Reports* | 13 | 264 | 10 | 4.6 |
| 6 | *Gut* | 10 | 1063 | 9 | 24.5 |
| 7 | *World Journal of Gastroenterology* | 9 | 84 | 4 | 4.3 |
| 8 | *Developmental Biology* | 8 | 370 | 7 | 2.7 |
| 9 | *Glia* | 8 | 479 | 7 | 6.2 |
| 10 | *Journal of Physiology-London* | 8 | 345 | 7 | 5.5 |

IF, impact factor.
