# Supplementary figures and images for: Global research status and trends of enteric glia: a bibliometric analysis
Source: Front Pharmacol. 2024 May 24;15:1403767. doi: 10.3389/fphar.2024.1403767 (PMC11157232; doi:10.3389/fphar.2024.1403767)

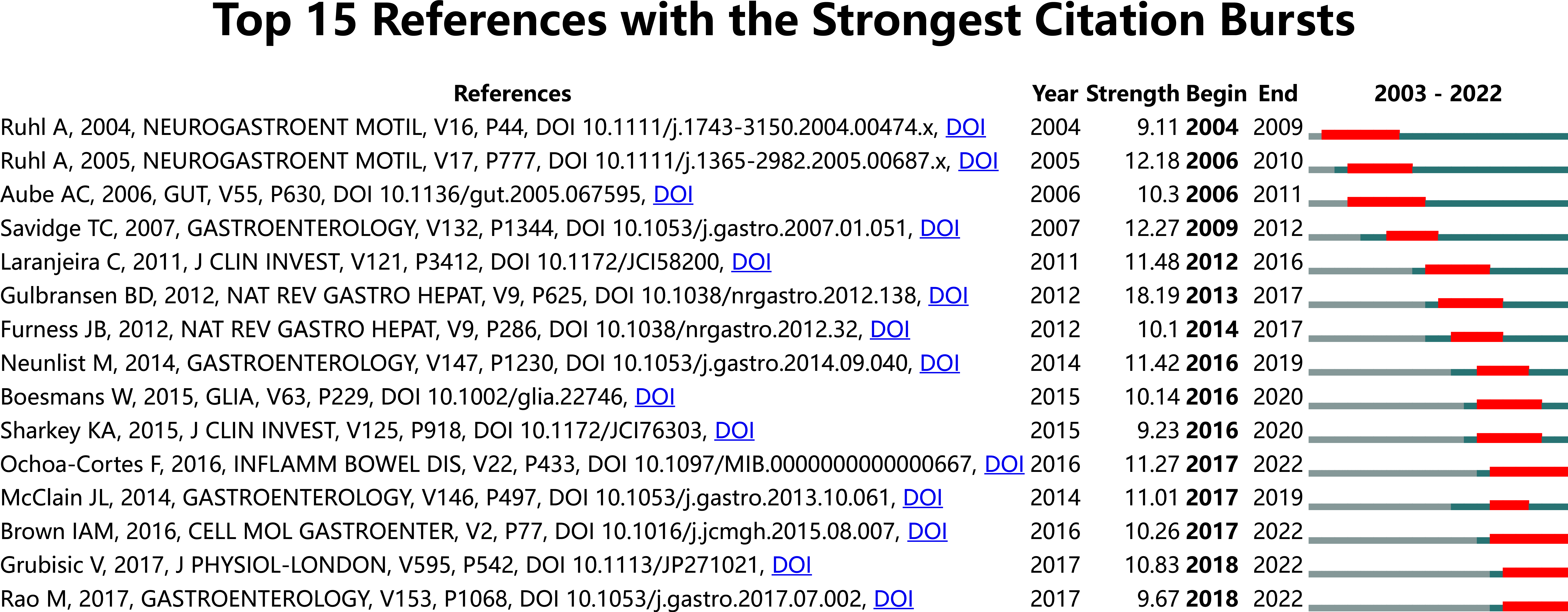

Supplement: Supplementary file 3 [file Image1.PNG]
